# The effect of orthokeratology treatment zone decentration on myopia progression

**DOI:** 10.1186/s12886-022-02310-4

**Published:** 2022-02-15

**Authors:** Lu Sun, Zheng-Xuan Li, Yun Chen, Zhi-Qiang He, Hong-Xin Song

**Affiliations:** 1 Beijing Tongren Eye Center, Beijing Institute of Ophthalmology, Beijing Tongren Hospital, Capital Medical University, Beijing Key Laboratory of Ophthalmology and visual Sciences, National Engineering Research Center for Ophthalmology, #1 Dong Jiao Min Xiang, Beijing, 100730 China; 2 Key Laboratory of Universal Wireless Communications, Ministry of Education, Beijing University of Posts and Telecommunications, Beijing, 100876 China

**Keywords:** Myopia, Axial length elongation, Decentration of Ortho-K lens

## Abstract

**Background:**

This study aimed to compare the changes in the axial length (AL) in myopic children that wear centered and decentered orthokeratology (Ortho-K).

**Methods:**

This retrospective study included 217 subjects who were treated with an Ortho-K lens for >12 months. The subjects were divided into three groups based on the magnitude of the Ortho-K lens treatment zone decentration: mildly, moderately, and severely decentered groups. Distance and direction of treatment zone decentration were calculated using software that was developed in-house. The AL changes in different groups were compared.

**Results:**

Based on the distance of the treatment zone decentration, 65 children (65 eyes) were included in the mildly decentered group, 114 children (114 eyes) in the moderately decentered group, and 38 children (38 eyes) in the severely decentered group. The mean decentration distance in the three groups was 0.35 ± 0.11 mm, 0.71 ± 0.13 mm, and 1.21 ± 0.22 mm, respectively. The mean AL increase in the three groups after 12 months of Ortho-K lens wear was 0.24 ± 0.21 mm, 0.23 ± 0.18 mm, and 0.19 ± 0.20 mm, respectively. There were no significant differences in AL changes among the three groups.

**Conclusions:**

Ortho-K lens decentration is common in clinical practice. The AL change after Ortho-K lens wear was not significantly different in subjects with different magnitudes of Ortho-K lens decentration. Fitting the Ortho-K lens in the properly centered zone is recommended to ensure the safety of Ortho-K lens wear and to maintain visual quality.

## Background

Myopia (which is known as near-sightedness) is an abnormal eye condition, in which images from distant objects are focused in front of the retina, which results in blurred vision. Myopia is a major cause of visual impairment, and its prevalence is increasing globally. It has been estimated that the population of myopic patients will rise to approximately 5 billion globally by 2050 [1]. Myopia occurs due to ocular length elongation, which might be caused by sclera stretching [2]. High myopia has a higher risk to develop many ocular diseases, such as cataracts, glaucoma, macular degeneration, and choroid retinal complications [3].

Orthokeratology (Ortho-K) is a popular, non-surgical method that has been widely used to correct myopia and can control myopia progression in adolescents [4–6]. An Ortho-K lens exerts a positive push pressure on the central cornea and produces a negative suction on the peripheral cornea, which results in the redistribution of corneal epithelial cells to the periphery and thinning of the central cornea [7]. Corneal changes that are caused by Ortho-K lens allow light to be focused on the mid-peripheral retina and macula, and the peripheral light is focused in front of the peripheral retina (myopia defocus) [8]. Recently, numerous clinical studies confirmed that Ortho-K lens wear at night significantly slowed the progression of myopia and resulted in elongation of the axial length (AL) [9–14].

The ideal shape of the cornea after Ortho-K lens wear includes a centrally flattened zone and a bulls-eye pattern on the corneal tangential difference map. However, the center of the Ortho-K lens treatment zone is often inconsistent with the center of the pupil in Ortho-K lens wearers. This Ortho-K lens decentration might be caused by multiple factors, which include eyelid tension, corneal astigmatism, or Ortho-K lens movements [15]. Ortho-K lens decentration is a major cause of post-treatment visual quality defects, which include halo, glare, reduced visual acuity, and increased aberration [16].

Controversial conclusions remain in the literature on the effect of Ortho-K lens decentration on the progression of myopia. For example, Wang et al. [17] found that in the absence of obvious corneal complications, decentered Ortho-K lens fitting was more effective at delaying myopia progression compared with centered Ortho-K lens fitting. Lau et al. found that higher-order aberration was negatively correlated with axial elongation [18]. Hiraoka et al. reported that coma-like aberrations increased with the magnitude of treatment zone decentration [16]. However, Santodomingo-Rubido et al. found that Ortho-K lens wear in the short and long-term caused significant changes in corneal aberrations: however, this was not correlated with the changes in AL elongation after 2 years follow-up [19].

This study aims to analyze the effect of centered and decentered Ortho-K lens wear on myopia. In addition, the AL changes after Ortho-K lens wear for>12 months were compared between groups with different magnitudes of Ortho-K lens treatment zone decentration.

## Methods

### Subjects

This retrospective study included 217 subjects who wore Ortho-K lens for >12 months in the myopic control outpatient clinic of Beijing Tongren Hospital (Beijing, China) from January 2017 to December 2019. This study adhered to the tenets of the Declaration of Helsinki and was approved by the Ethics Committee of Beijing Tongren Hospital. Written informed consent form was obtained from all subjects and their guardians.

The inclusion criteria were: (1) a spher ical equivalent refractive error (SER) of <–6.00 diopter sphere (DS); and (2) a best-corrected visual acuity (BCVA) of 20/20 or better after the removal of the lens. To eliminate the between-eye correlation, the right eyes of the subjects were included in this study. Subjects who had strabismus, systemic diseases, or complications associated with Ortho-K lens wear, such as glare or corneal epithelial defect, were excluded from this study.

### Procedures

All the subjects underwent comprehensive baseline ocular examinations, which included slit-lamp examination, uncorrected visual acuity measurement, BCVA obtained with manifest refraction, fundus examination, and AL measurement. Cycloplegic refraction was performed at the first visit, and cycloplegia was achieved by applying four drops of compound tropicamide eye drops (0.5% tropicamide and 0.5% neo-synephrine; Santen, Japan) with 5 m intervals. Then, 10 minutes after the application of the fourth drop, autorefraction was performed three times (TOPCON, Japan, model: KR-8100) and the mean value was calculated. AL was measured using a noncontact partial coherent interferometry (IOL-Master 500; Carl Zeiss, Germany). Corneal Topographer (Medmont E300, Medmont International PTY LTD, Australia) was used to obtain the corneal parameters by the same professional technician.

After Ortho-K lens fitting, all subjects were advised to wear their lenses every night for ≥8 consecutive h. Examinations were performed 1 day, 1 week, 1 month, and every 3 months after Ortho-K lens wear. At each visit, all subjects received a detailed slit-lamp examination, BCVA measurement, and corneal topography. AL measurements were carried out every 6 months.

### Determination of treatment zone decentration

**Fig. 1:**
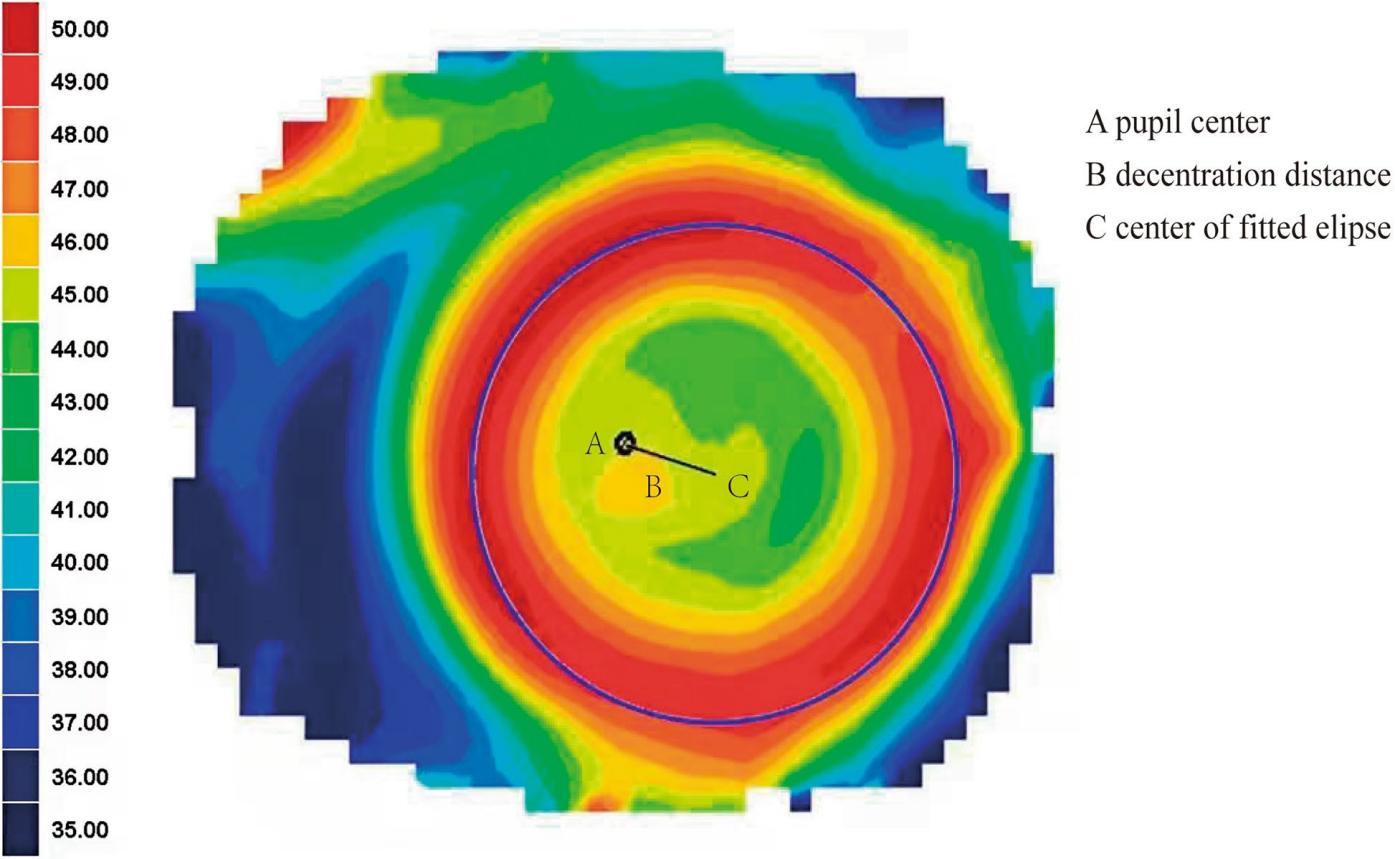
A computer program developed in-house was adopted to measure the distance of ortho-K lens decentration for each subject. The blue ellipse was considered to be the fitting ellipse. The black line represents the decentration distance between the pupil center and the center of the fitted ellipse

A Corneal Topographer (Medmont E300) was used to collect the corneal surface data from the subjects and the tangential power map of corneal topography after 1 month of Ortho-K lens wear was used for analysis. An in-house developed computer program (Python) was used to measure the distance of the Ortho-K lens decentration. The data on the tangential radius of curvature and its coordinates were obtained from a Medmont Topographer. Then, the data on the tangential radius of curvature were converted into data on the tangential diopter, and the noise points generated during the acquisition process were removed by mean filtering. The sets of all maximum points in each direction (300 directions) of the tangential diopter were clustered with the K-means algorithm, and all the points in the decentered zone were identified. The data points obtained with the K-means algorithm were fitted to obtain a fitted ellipse. The distance between the center of the ellipse and the center of the pupil was defined as the decentration distance, and the severity of decentration was classified based on the decentration distance. Figure 1 shows the corneal topography data 1 month after Ortho-K lens wear. The red circle indicates the treatment zone of the Ortho-K lens. An ellipse was fitted on top of the treatment zone and the center of the ellipse was defined as the center of the treatment zone. The black line represents the decentration distance between the pupil center and the center of the fitted ellipses.

The definition of the magnitude of the treatment zone decentration proposed by Tsai and Lin was used in this study [20]. The subjects were divided into three groups based on the decentration distance: mildly decentered group (decentration distance <0.5 mm), moderately decentered group (decentration distance >0.5 mm and <1.0 mm), and severely decentered group (decentration distance >1.0 mm).

### Statistical analysis

Continuous variables were presented as mean ± standard deviation (interquartile range (IQR) where appropriate. Categorical variables were presented as numbers and percentages (%). The Shapiro-Wilk test was performed to examine if the continuous variables were normally distributed. Group differences in baseline characteristics and ALs were analyzed using the Kruskal–Wallis H test or one-way analysis of variance (ANOVA). To compare the changes in AL in the three groups, covariance analysis with the covariates adjusted was conducted. Multiple linear regression was used to analyze the associations between potential variables and AL. All statistical analyses were performed using SPSS software (26.0; SPSS Inc., Chicago, IL) with the significance level established at two-sided *p*<0.05.

## Results

**Fig. 2:**
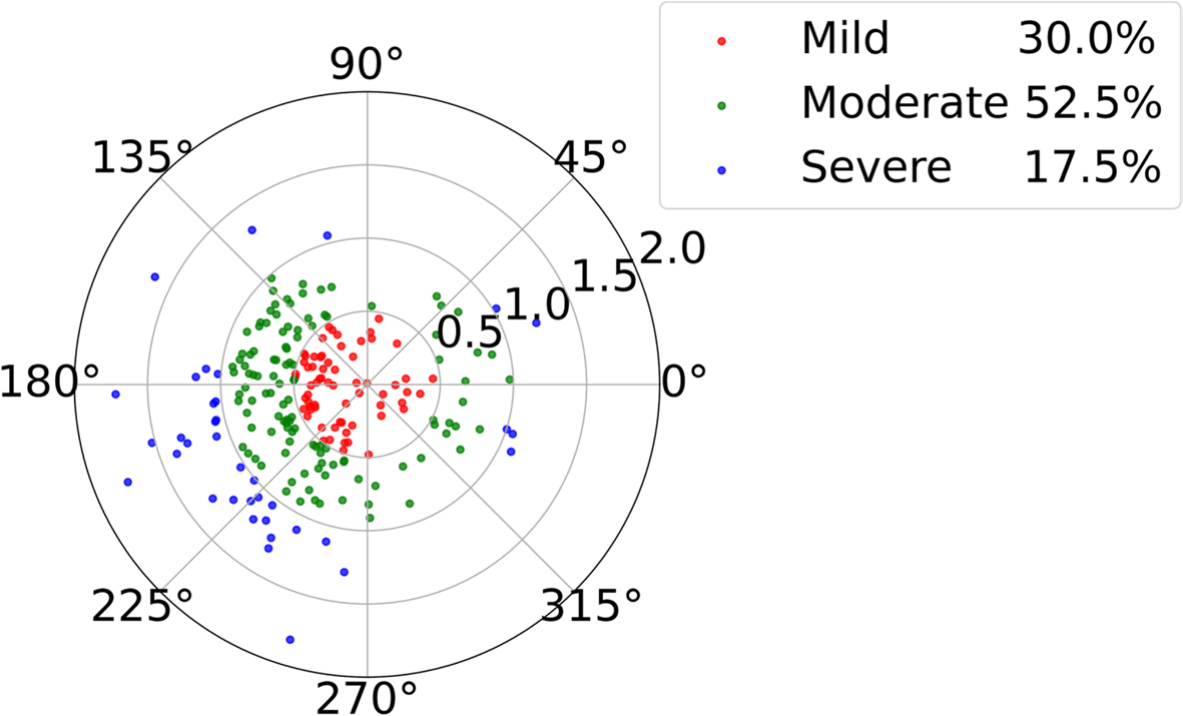
Overview of lens decentration for all subjects. Overview of treatment zone Ortho-K lens decentration. The range between 0 and 360° was similar to the meridian degree set on a corneal topography map. Each circle outlines the distances of 0.5 and 1.0 mm to the corneal vertex normal

Throughout this study, all subjects maintained a visual acuity >20/20 or better. After 1-month Ortho-K lens wear, the treatment zone decentration distance in the mildly, moderately, and severely decentered groups was 0.35 ± 0.11 mm (0.01–0.49 mm), 0.71 ± 0.13 mm (0.50–0.97 mm), and 1.21 ± 0.22 mm (1.00–1.81 mm), respectively. For the horizontal displacement, temporal decentration was observed in 173 eyes (79.7%). For the vertical displacement, inferior decentration was observed in 123 eyes (56.7%). For the overall displacement, inferotemporal decentration was the most commonly observed decentration and occurred in 97 eyes (44.7%). Figure 2 shows the overview of lens decentration that was observed in all subjects.

In total, there were 65 subjects (31 males and 34 females) in the mildly decentered group, with a mean age of 12.49 ± 1.75 years (10–19 years), 114 subjects (56 males and 58 females) in the moderately decentered group, with a mean age of 12.45 ± 1.58 years (10–17 years), and 38 subjects (15 males and 23 females) in the severely decentered group, with a mean age of 12.26 ± 2.10 years (9–19 years). No statistical difference was found in age, gender, baseline AL, spherical refractive error, astigmatism, equivalent e-value, flat K, steep K, and deviated angle (*p*>0.05, Table 1).

**Table 1:** Biometric measurements of study subjects

Variables mean ± SD, n (%)	All *n*= 217	Mildly *n* = 65	Moderately *n* = 114	Severely *n* = 38	*P-*value^a^
Age (years)	12.43 ± 1.73	12.49 ± 1.75	12.45 ± 1.58	12.26 ± 2.10	0.621
Gender (male)	102 (47)	31 (48)	56 (49)	15 (40)	0.582
SER	-3.10 ± 1.20	-2.95 ± 1.30	-3.10 ± 1.20	-3.30 ± 1.04	0.328
SRE	-3.0 ± 1.12	-2.86 ± 1.16	-3.01 ± 1.12	-3.19 ± 1.04	0.285
RA	90 (41)	28 (43)	48 (42)	15 (39)	0.937
Baseline AL	24.81 ± 0.83	24.75 ± 0.90	24.80 ± 0.80	24.97 ± 0.77	0.417
Flat K	42.70 ± 1.28	42.81 ± 1.49	42.69 ± 1.19	42.51 ± 1.18	0.530
Steep K	43.66 ± 1.39	43.74 ± 1.56	43.68 ± 1.33	43.45 ± 1.33	0.660
DD, mm	0.69 ± 0.32	0.35 ± 0.11	0.71 ± 0.13	1.21 ± 0.22	<0.001
DA	192.43 ± 74.33	189.89 ± 76.90	188.03 ± 74.17	209.94 ± 69.58	0.111
Flat E	0.59 ± 0.14	0.61 ± 0.12	0.59 ± 0.15	0.56 ± 0.15	0.324

*SER* Spherical equivalent refractive error, *SRE* Spherical refractive error, *RA* Regular astigmatism, *AL* Axial length, *DD* Decentered distance, *DA* Decentered Angle

^a^According to the distribution, Kruskal–Wallis H test or ANOVA was adopted to compare characteristics among the three groups.

After 12 months of Ortho-K lens wear, the average AL in the mildly decentered group increased from 24.75 ± 0.90 mm to 24.99 ± 0.88 mm, with an increase of 0.24 ± 0.21 mm. The subjects in the moderately decentered group had AL changes from 24.80 ± 0.80 mm to 25.03 ± 0.76 mm with an increase of 0.23 ± 0.18 mm, and the subjects in the severely decentered group have AL changes from 24.97 ± 0.77 mm to 25.16 ± 0.78 mm with an increase of 0.19 ± 0.20 mm. One-way ANOVA and before and after the test were used to analyze AL changes in subjects with different magnitudes of decentration after 12 months of Ortho-K wear. There were no significant differences in the AL change among the mildly, moderately, and severely decentered groups (*p* = 0.486; Table 2).

**Table 2:** Changes in AL from baseline to 12 months after Ortho-K lens wear

Group	Number	Baseline (mm)	12 months (mm)	AL change (mm)
Mildly	65	24.75 ± 0.90	24.99 ± 0.88	0.24 ± 0.21
Moderately	114	24.80 ± 0.80	25.03 ± 0.76	0.23 ± 0.18
Severely	38	24.97± 0.77	25.16 ± 0.78	0.19 ± 0.20
*p-*value		/	/	0.486

*AL* Axial length

Multiple linear regression analysis was used to analyze risk factors that affected the amplitude of AL changes, which included age, gender, spherical refractive error, astigmatism, and corneal curvature. Age was the only risk factor for AL elongation (*p*<0.001). On average, the overall AL change decreased by 0.037 mm per year. However, there was no significant difference in the age-associated AL change between the moderately decentered group and the severely decentered group (*p*>0.05, Table 3).

**Table 3:** Multiple Linear regression of AL changes from baseline to 12 months after Ortho-K lens wear

Variable	Estimate	Standard error	t value	*P-*value
Intercept	1.1934	0.447	2.67	0.008
Age (years)	-0.037	0.007	-4.97	<0.001
Gender (male)	-0.045	0.027	-1.69	0.092
Decentration (ref = mild)				
Moderately	-0.007	0.028	-0.25	0.807
Severely	-0.049	0.037	-1.34	0.183
Baseline AL	-0.017	0.020	-0.88	0.379
SRE	0.013	0.012	1.11	0.267
RA	0.003	0.026	0.10	0.917
Flat K	-0.001	0.024	-0.02	0.982
Steep K	0.009	0.022	0.411	0.681

*AL* Axial length

Covariance analysis showed that there was no statistical difference in the AL change in subjects with different magnitudes of decentration (*p* = 0.37, Table 4).

**Table 4:** Covariance analysis for changes in AL in children with mildly, moderately, and severely decentered groups

Variable	Mean square	*F* value	*P-*value
Age (years)	0.800	24.69	<0.001
Gender (male)	0.093	2.87	0.092
Decentration (mildly, moderately, severely)	0.032	1.00	0.370
Baseline AL	0.025	0.78	0.379
SRE	0.040	1.24	0.267
Astigmatism	0.000	0.01	0.917

*AL* Axial length

## Discussion

Currently, >1.5 million teenagers in China use Ortho-K lenses [21]. Although the safety of short and long-term Ortho-K lens wear has been demonstrated [5, 21–25], the treatment zone decentration of the Ortho-K lens is common during Ortho-K treatment. Some studies reported that approximately 50% of Ortho-K lens wearers experienced different magnitudes of decentration [16, 26]. However, the effect of Ortho-K lens decentration on AL changes remains unclear. In this study, Ortho-K lens decentration did not significantly cause AL changes in children.

In this study, a computer program that was developed in-house was used to measure the distance subjectively and accurately between the center of the Ortho-K lens treatment zone and the center of the pupil. Since the corneal shaping process that is caused by Ortho-K lens wear is complete and stable 7–10 days after wearing the contact lens [27], the distance of decentration was calculated 30 days after Ortho-K lens wear, using the corneal topography map.

In this study, the multi-factor linear regression results showed that age was an influential factor for AL elongation (*p*<0.001), and the average AL change decreased by 0.037 mm per year. This result agreed with a previous study that was conducted in Singapore, which reported that the annual AL increase in myopia decreased with age [28].

However, the effect of Ortho-K lens decentration on AL changes in myopic children remains controversial. Wang and Yang reported that in a self-control study, AL changes in an eye with off-center Ortho-K lens was less than that in an eye with a centered Ortho-K lens in children who wore an Ortho-K lens in both eyes [17]. Chen et al. [29] found that the decentration in the treatment zone slightly reduced the increase in AL. However, in this study, there were no significant AL changes in subjects with different degrees of Ortho-K lens decentration. The discrepancy between different studies might be due to different conditions, such as the follow-up time or the sample size. Although Ortho-K lens can effectively control the progression of myopia, the mechanisms remain largely unclear. Ortho-K lens slows the development of myopia, possibly because Ortho-K lens causes the central cornea flatten and increases the steepness of the mid-peripheral cornea, which can reduce the hyperopic defocus on the peripheral retina, thereby reducing the visual feedback for AL elongation [30–32]. When the pupil is closer to the edge of the treatment area, the peripheral area will experience more myopic defocus, or increase the total peripheral myopic defocus [17, 33]. This may explain why the lens decentration can reduce the growth of AL better. In addition, Ortho-K lens treatment zone decentration significantly affects corneal and whole eye ocular aberrations [34]. Cho et al. [9] proposed that higher order aberrations induced by Ortho-K lens wear may be a stimulus for slowing eye growth. Moreover, Oshika et al. reported that coma-like aberration of the cornea was significantly positively correlated with the magnitude of apparent accommodation in pseudophakic eyes [35]. Increases in Ortho-K lens treatment zone decentration may contribute to increase in corneal pseudo-accommodation, which might influence AL elongation [29].

Chen et al. found that baseline SER, astigmatism, e-value, and flat K did not affect Ortho-K lens decentration [15], which agreed with the finding of this study (Table 3). For the direction of decentration, temporal and inferior decentration occurred most frequently in all three groups, which agreed with the results previously reported [16, 26]. The temporal cornea is flatter than the nasal cornea after Ortho-K lens wear [36]. Therefore, the asymmetry between the nasal and temporal cornea might cause Ortho-K lens decentration. In addition, the unequal alignment of the Ortho-K lens along the main meridian could affect the stability of the Ortho-K lens, and therefore, might increase the possibility of Ortho-K lens decentration [37]. In addition, external forces, such as the eyelid force, which are due to large differences in the cornea central curvature in the inferior and superior meridian, might push the Ortho-K lens down, resulting in inferior decentration [38].

Severe Ortho-K lens decentration can cause corneal problems, such as staining and indentation in the corneal epithelium [29]. In addition, severe Ortho-K lens decentration increases high-order aberration, reduces contrast sensitivity, and causes visual interference, glare, halo, and other complications, which might compromise the efficacy of Ortho-K lens treatment [16, 39]. The effect of Ortho-K lens decentration on the progression of myopia remains controversial. Long-term studies are required to demonstrate the effect of Ortho-K lens treatment zone decentration. Therefore, in clinical practice, increasing the lens decentration on purpose is not encouraged.

This study has some limitations. First, since the peripheral defocus was not measured in the moderately and severely decentered groups, it is unknown if there is more myopia defocus in the peripheral areas when the pupil is closer to the edge of the treatment zone. Second, this study did not reveal a statistical difference in the progression of myopia between the mildly decentered group and the moderately and severely decentered groups. This might be due to a relatively short-term period of Ortho-K lens wear (12 months) and insufficient sample size. In future studies, to explore the relationship between peripheral defocus and myopia control effect, a quantitative algorithm should be established to calculate the peripheral defocus after Ortho-K wear.

## Conclusions

No statistical difference in the effect of myopia control was found among the mildly, moderately, and severely decentered groups. To maintain visual quality and ensure the safety of Ortho-K lens wear, fitting an Ortho-K lens in the central position is recommended.

## Data Availability

The datasets used and/or analyzed during the current study are available from the corresponding author on reasonable request.

## Abbreviations

Ortho-K: Orthokeratology
AL: Axial length;
SER: Spherical equivalent refractive error
DS: Diopter sphere
BCVA: Best corrected visual acuity
